# The Evolution of the Bacterial Luciferase Gene Cassette (*lux*) as a Real-Time Bioreporter

**DOI:** 10.3390/s120100732

**Published:** 2012-01-11

**Authors:** Dan Close, Tingting Xu, Abby Smartt, Alexandra Rogers, Robert Crossley, Sarah Price, Steven Ripp, Gary Sayler

**Affiliations:** 1 Oak Ridge National Laboratory, The Joint Institute for Biological Sciences, 676 Dabney Hall, Knoxville, TN 37996, USA; E-Mail: dclose@utk.edu; 2 The Center for Environmental Biotechnology,The University of Tennessee, 676 Dabney Hall, Knoxville, TN 37996, USA; E-Mails: txu2@utk.edu (T.X.); asmartt@utk.edu (A.S.); aroger24@utk.edu (A.R.); rcrossl1@utk.edu (R.C.); sprice12@utk.edu (S.P.); saripp@utk.edu (S.R.)

**Keywords:** mammalian cells, bacterial luciferase (*lux*), bioreporter, biosensor, cell culture, small animal models

## Abstract

The bacterial luciferase gene cassette (*lux*) is unique among bioluminescent bioreporter systems due to its ability to synthesize and/or scavenge all of the substrate compounds required for its production of light. As a result, the *lux* system has the unique ability to autonomously produce a luminescent signal, either continuously or in response to the presence of a specific trigger, across a wide array of organismal hosts. While originally employed extensively as a bacterial bioreporter system for the detection of specific chemical signals in environmental samples, the use of *lux* as a bioreporter technology has continuously expanded over the last 30 years to include expression in eukaryotic cells such as *Saccharomyces cerevisiae* and even human cell lines as well. Under these conditions, the *lux* system has been developed for use as a biomedical detection tool for toxicity screening and visualization of tumors in small animal models. As the technologies for *lux* signal detection continue to improve, it is poised to become one of the first fully implantable detection systems for intra-organismal optical detection through direct marriage to an implantable photon-detecting digital chip. This review presents the basic biochemical background that allows the *lux* system to continuously autobioluminesce and highlights the important milestones in the use of *lux*-based bioreporters as they have evolved from chemical detection platforms in prokaryotic bacteria to rodent-based tumorigenesis study targets. In addition, the future of *lux* imaging using integrated circuit microluminometry to image directly within a living host in real-time will be introduced and its role in the development of dose/response therapeutic systems will be highlighted.

## 1. Introduction

Bacterial bioluminescence, commonly known as the *lux* reaction, is the most widely distributed luminescent mechanism on the planet [1] and, although this process of bacterial light production has been observed for centuries, it was not until the mid 1900s that it began to be evaluated scientifically [2,3]. Beginning in the 1980s, after several decades of research, the understanding of this system became advanced enough that it was possible to exogenously express the full gene cassette, comprised of five genes (*luxCDABE*), in alternative host organisms such as *Escherichia coli* [4]. As researcher’s understanding of the biochemistry behind the *lux* reaction continued to be refined, and genetic manipulation techniques improved, it soon became possible to exploit this cassette as a reporter system across a wide variety of bacterial species for an extremely diverse set of monitoring objectives.

Following the success of these myriad *lux*-based bacterial bioreporters, attempts were made to incorporate the system into eukaryotic organisms in order to expand the *lux* system’s usefulness as a reporter. While initially expression of the bacterial genes was unsuccessful, through rearrangement of the *lux* cassette gene expression pattern and improvement of expression efficiency via codon-optimization and the addition of specialized linker regions, these hurdles were overcome and the *lux* reaction was demonstrated to occur in the lower eukaryote *Saccharomyces cerevisiae* [5]. Building upon this early success of eukaryotic expression, the *luxAB* genes were then further engineered to express in a human cell line, leading to the emergence of the *lux* system as a truly multifunctional reporter system similar to the more commonly employed firefly luciferase system [6].

Recently the *lux* system has undergone another substantial improvement, as it has been demonstrated that the full cassette can be optimized in a similar manner to the *luxAB* genes in order to promote fully autonomous bioluminescent production in a human cell line without the need to exogenously supplement a chemical substrate [7]. This review will highlight the development of the *lux* cassette from a curiosity observed in marine bacteria, through its extensive use as a bacterial bioreporter system and modification for expression in eukaryotic organisms, up to its recent demonstration as the only fully autonomous, substrate-free bioluminescent reporter system available in the eukaryotic host background. The unique, autonomous nature of the *lux* cassette will also be reviewed in light of the development of advanced photon detection hardware, detailing the future directions of *lux* development and its potential for biomedical as well as basic research applications.

### Wild-Type lux Background

**Figure 1.**
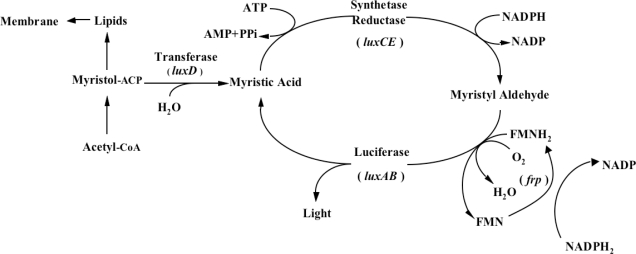
The *luxCDABEfrp* genes work synergistically with endogenous myristic acid, FMN, and O_2_ to generate a bioluminescent signal. The *frp* gene is not found in all organisms expressing the remaining *lux* genes. Originally published in and used with permission from [1].

One does not have to look very far to see the glow of naturally bioluminescent organisms. On land bioluminescence is most commonly observed in the glow of fungi growing on decaying wood or from insects displaying their luminescent signal after dusk, while in marine environments bioluminescence is most commonly observed in single celled bacteria that are found either living freely or in symbiosis with larger hosts. It is these bioluminescent bacteria that are the most abundant and widely distributed of the light emitting organisms on Earth and they can be found in both aquatic (freshwater and marine) and terrestrial environments. Despite the widespread prevalence of bacterial bioluminescence, however, the majority of these organisms are classified into just three genera: *Vibrio*, *Photobacterium*, and *Photorhabdus* (*Xenorhabdus*) [1]. Although they are viable as free-living bacteria, these organisms are most commonly observed in symbiosis with a larger host. There is still no consensus as to the evolutionary benefit of bioluminescent production, however, in general it is theorized that the production of light can aid in the consumption of free living bacteria by higher trophic organisms, transferring them to a more controlled, nutrient rich habitat inside the host, or that, likewise, symbiotic bacteria can aid their hosts through the production of light that attracts prey, aids in camouflage, or attracts mates, in return for the shelter and nutrients provided by living within the body of the host organism [8]. Regardless of the reasons, the genetic system employed for the generation of bioluminescence is well conserved across all known bioluminescent bacteria. The luciferase protein is a heterodimer formed by the *luxA* and *luxB* gene products. The *luxC*, *luxD*, and *luxE* gene products encode for a reductase, transferase, and synthase respectively, that work together in a single complex to generate an aldehyde substrate for the bioluminescent reaction. In some species, there is an additional gene, *frp*, that functions as a flavin reductase to aid in the regeneration of the required FMNH_2_ substrate. Together with molecular oxygen, these components are all that are required to produce a bioluminescent signal [9] (Figure 1).

**Figure 2.**
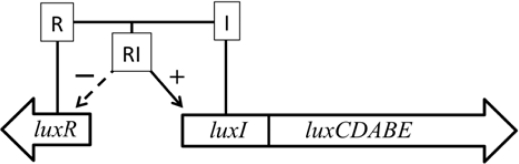
In some species, an autoinducer (I) is produced by the *luxI* gene and a transcriptional activator (R) is produced by the *luxR* gene. When these two components interact (RI), they enhance production of the *luxCDABE* genes and inhibit further expression of the *luxR* gene. This feedback system serves to regulate bioluminescent production in these organisms.

In addition, some marine species have additional genes that govern the expression of the remainder of the operon. The *luxI* and *luxR* genes function as an autoinducer and transcriptional activator (Figure 2), allowing the bioluminescent bacteria to participate in quorum sensing, therefore producing a bioluminescent signal only at high population densities [10]. This allows the production of light to be regulated and only expressed when it is beneficial to the organism, without unduly consuming metabolic energy.

## 2. Bacterial *lux*-Based Reporter Systems

### 2.1. First Examples of Transgenic lux Expression

Even before the mechanisms responsible for bioluminescence were completely explored in the organisms that naturally express it, the *lux* system had gained attention in regards to its potential as a reporter because of the facile detection and quantification of visible light produced by the Lux proteins. Work therefore quickly began to introduce the *lux* genes into non-luminescent bacterial species to determine if the modified organisms could acquire the light-emitting phenotype. These initial attempts to isolate and exogenously express the *lux* genes in non-native hosts such as *E. coli* were driven primarily by the desire to understand the organization, regulation, and function of the genetic components responsible for bioluminescence. The first major advancement in these investigations occurred in 1982 when Belas *et al*. [4] demonstrated bioluminescent production from recombinant *E. coli* containing the *Vibrio harveyi luxA* and *luxB* genes upon addition of exogenous aldehyde. In less than one year following this successful demonstration, the entire *lux* cassette (*luxCDABE*), together with its associated regulatory genes, was isolated from *V. fischeri* and introduced into *E. coli* [11], resulting in the first recombinant strain capable of emitting light without aldehyde supplementation. This process was soon repeated using the *Photorhabdus luminescens* genes, demonstrating the universal nature of the operon [12,13]. Although these efforts focused primarily at interrogating the functions of the individual *lux* genes, investigators began to appreciate the sensitivity, ease of detection, and non-destructive features the *lux* system offered as a bioluminescent reporter. The practical nonexistence of *in vivo* bioluminescence from bacteria not expressing the *lux* genes provides a high signal-to-noise ratio for the recombinant *lux* reporter system and the fast turnover rate of the bacterial luciferase enzyme allows for rapid light production. Taken together, these attributes have allowed the development of bacterial bioreporters that can be continuously monitored in a near real-time fashion. In addition, because the resulting bioluminescence is emitted in an autonomous manner, these reporters can be employed in a high throughput manner with very low cost, making them attractive options for a large number of biomonitoring applications.

### 2.2. Development of lux as a Method for Visualizing Gene Expression

The first use of *lux* as a biomonitoring technology came soon after its transgenic expression in *E. coli*, when Enbreghet *et al*. [14] fused the *lux* cassette to an inducible promoter that could be used to monitor gene expression *in vivo*. Using this experimental design it became possible to monitor autonomous bioluminescence as an indicator for the transcriptional activity of a promoter of interest. Using this method, the first major targets of study were the *E. coli lac* and *ara* promoters and it was discovered that upon IPTG or arabinose induction, light production in hosts expressing *lux* fusions increased between 600 to 1,000-fold. Following these reports the *lux* system was used to monitor regulation of the lateral flagella genes in *Vibrio parahaemolyticus* [14,15], providing its first demonstration in a previously uncharacterized system. These applications represented a significant shift in the way gene expression was investigated because, unlike traditional biochemical assays using enzymatic reporters, the bioluminescent signal from the *lux* genes could be easily detected and measured with high sensitivity without cell perturbation. This allowed the same sample to be continuously monitored, thus revealing the dynamics of gene expression through changes in bioluminescence over time. This new method was therefore capable of generating data that could not previously be generated.

### 2.3. lux-Based Bioluminescence as a Tool for Cellular Population Monitoring

While the Lux proteins do not require exogenous substrate addition, their function does require continued access to the molecular oxygen, FMNH_2_, and aldehyde co-substrates. For this reason, their bioluminescence can only be detected in actively growing cells. This knowledge, combined with the discovery that *lux* bioluminescent output is proportionally correlated to the number of cells present, has therefore been used as a simple, sensitive, and non-destructive means for *in situ* bacterial monitoring. This was first demonstrated by Shaw *et al*. [16] in 1986 when constitutively expressed *V. fischeri luxCDABE* genes were introduced into the phytopathogen *Xanthomonas campestris*, and their subsequent invasion of a cauliflower leaf was visualized. Similarly, de Weger and colleagues [17] were successfully able to detect *luxCDABE*-labeled *Pseudomonas fluorescens* in the rhizosphere of soybean roots using the same technique. Additionally, through the use of a *lux*-based system rather than an enzymatic reporter, it was possible for these researchers to achieve detection limits three orders of magnitude lower than what was previously possible, leading to improved signal detection. These early examples highlighted the application of *lux*-based bioluminescence as a rapid, simple and sensitive tool for *in situ* detection of living bacteria and established the foundation for future research using *lux* to monitor genetically engineered microorganisms. In perhaps the most notable use of the *lux* genes for tracking a cellular population, a *P. fluorescens* strain was transformed with the *lux* genes and used for the first bioremediation-related environmental field release of a genetically engineered microorganism in 1996.

**Figure 3.**
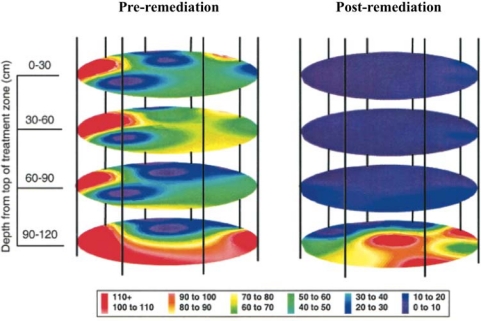
Using bioluminescent bacteria, Ripp *et al*. were able to track both the presence of the genetically modified organisms as well as the their effectiveness in degrading naphthalene over time. Naphthalene concentrations are shown in ppm. Adapted and used with permission from [18].

This release was approved by the Environmental Protection Agency to determine the efficiency of bioremediation process monitoring through inoculation of the bioluminescent strain directly into contaminated soil and to determine its ability to monitor the bioremediation of polycyclic aromatic hydrocarbons [18]. By placing the *lux* genes under the control of promoters in the naphthalene degradation pathway, it was possible to monitor their bioluminescent output as a measure of naphthalene contamination in the soil [19]. Using a combination of bioluminescent and traditional culture based detection methods, the release area was monitored for two years after the release of bioluminescent *P. fluorescens*. Over this time, regular sampling was performed to track the amount of bacteria present in the soil, as well as the amount of bioluminescence produced, which were indicative of organism presence and naphthalene degradation, respectively (Figure 3). Based on culture detection methods, the bioluminescent *P. fluorescens* persisted in both contaminated and non-contaminated soils, decaying at similar rates and producing similar colony counts [18]. The long term nature and difficulty in remote monitoring of bacterial populations presented in this study illustrates how the unique properties of the *lux* operon can provide it with an advantage over its substrate requiring bioluminescent or UV stimulation requiring fluorescent counterparts. Because of its autonomous nature, the *lux*-tagged *P. fluorescens* could be continually surveyed for bioluminescent production, without the need for repeated stimulation to induce a reporter signal.

### 2.4. The Use of lux for Exogenous Target Detection

**Figure 4.**
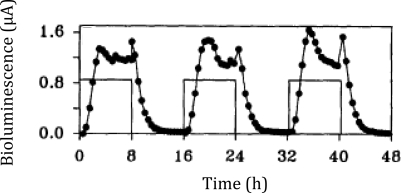
By treating with naphthalene over 8 h intervals (black boxes), King *et al*. were able to demonstrate a corresponding dose/response bioluminescent production (•) curve. Adapted and used with permission from [19].

Following the work that demonstrated how the *lux* cassette could be used as a tool for visualizing gene expression, it soon became clear that these genes could be adapted for use as a traditional bioreporter target through activation under specific, predetermined conditions as well. By expressing the *lux* cassette under the control of a promoter with a known inducer, the resultant bioluminescent emission could be used as an indicator for the presence of the given stimulus, and fluctuation of the bioluminescent signal could be interpreted as changes in the bioavailable concentration of the inducer compound. Building upon these ideas, the first use of bioluminescence for monitoring metabolic activity was demonstrated in *Pseudomonas putida* by Burlage *et al*. in 1990 [20]. Here, naphthalene degradation was monitored using a transcriptional fusion of the salicylate inducible *nah* promoter and the *luxCDABE* genes. Salicylate is an intermediate metabolite of naphthalene, which is eventually degraded to acetaldehyde and pyruvate in *Pseudomonas*. Therefore, naphthalene degradation could be correlated to the light emission upon induction with naphthalene-derived salicylate. The nondestructive nature of the *lux* system allowed for this analysis to occur in real time in a growing culture, providing continuous monitoring of naphthalene metabolism across various stages of growth. It was later determined by King *et al*. [19] that the bioluminescent signal was controlled in a dose/response fashion (Figure 4), therefore demonstrating its usefulness in determining contaminant levels in mixed environmental samples. This opened the door for a multitude of environmental bioreporters featuring *lux*, such as that developed by Applegate and colleagues that was used to monitor for water soluble benzene, toluene, ethylbenzene, and xylene (BTEX) compounds indicative of petroleum spills. This reporter, constructed by linking expression of the *lux* cassette to the toluene dioxygenase promoter, was capable of detecting as little as 30 μg of toluene/L in as quickly as 2 h and maintained its detection ability for over 100 generations without antibiotic selection [21].

Another common target for *lux*-based environmental sensing has been phenol. Notably, Abd-El-Haleem *et al*. [22] constructed one of the first *lux*-based phenol biosensors by inserting a *mopR*-like promoter fused to the *V. fischeri lux* cassette genes into *Acinetobacter* sp DF4. This reporter was capable of demonstrating a lower detection limit of 2.5 ppm in 4 h when exposed to phenol, and was only responsive to three of the ten phenol derivatives tested, suggesting that it was relatively specific as well. This is, however, not by any means the only *lux*-based phenol reporter to be developed. Davidov *et al*. [23] made extensive use of *recA* promoters fused to *lux* cassettes, with each of the reporters containing a slight variation in its promoter sequence, that were expressed either in *E. coli* or *Salmonella typhimurium* and using *lux* genes from either *V. fischeri* or *P. luminescens*. The most sensitive of these reporters was that expressing the *V. fischeri lux* genes in *E. coli*, which was capable of detecting 0.008 mg phenol/L in 2 h. This same construct, when expressed in *S. typhimurium* was also capable of detecting phenol in 2 h but required a minimum concentration of 16 mg phenol/L, demonstrating the differences in host phenol bioavailability.

### 2.5. Further Uses of lux as a Bacterial Bioreporter

As the popularity of the *lux* system has grown over the years, an increasing number of bacterial reporters have been leveraged for the detection of a wide variety of contaminants. While this review focuses only on the seminal examples of *lux*’s growth as a reporter system, a larger list of target compounds and detection limits of various bioreporters can be found in recent reviews [24,25] and Table 1.

## 3. Eukaryotic Expression of the *lux* Cassette

Despite its success as a bacterial bioreporter, widespread use of the *lux* system faced a major hurdle in that it was initially believed to be capable of expression only in prokaryotes. Although several attempts were made to express the *lux* genes in eukaryotic hosts, none of these made significant headway [74–76]. It would not be until 2003 that the first major achievement was documented with the demonstration of autonomous bioluminescence from the yeast *Saccharomyces cerevisiae* [5]. Following this breakthrough, the *lux* genes continued to be modified and improved for eukaryotic expression, later being developed into a reliable yeast-based bioassay tool and, eventually, becoming capable of expression in a human cell line [7], opening the door for continued development in the future.

### 3.1. lux Expression in Yeast

**Figure 5.**
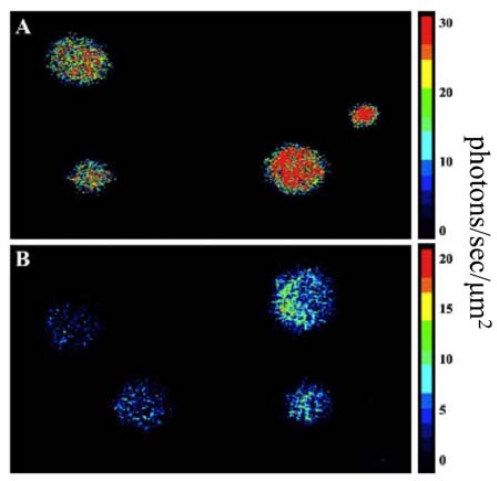
Comparison of (**A**) *S. cerevisiae* and (**B**) *E. coli* expressing the *luxCDABEfrp* genes. Used with permission from [5].

It was the demonstration of *lux* function in *S. cerevisiae* by Gupta *et al*. [5] in 2003 that marked the first time a eukaryotic organism successfully produced levels of bioluminescence comparable to prokaryotic *lux*-based bioreporters (Figure 5). To achieve this, Gupta and colleagues chose to express the *lux* genes from the terrestrial bacterium *P. luminescens* rather than those from the traditional marine organisms *V. harveyi* or *V. fischeri*. This was done because the resulting luciferase proteins from *P. luminescens* exhibit a higher thermal stability than those of their marine counterparts. To mimic the organization and expression of the *lux* genes found in prokaryotic organisms, the *luxA* and *luxB* genes were expressed from a single promoter and linked by an internal ribosomal entry site (IRES). Under this expression strategy it was determined that bioluminescence was 20 times greater than that reported for fused luciferases upon exposure to an n-decanal substrate. Building upon these findings, the remainder of the *lux* genes were incorporated using the same strategy, with a pair of genes linked by an IRES element and driven by a unique promoter. When expressed concurrently this design was capable of producing an easily detectable bioluminescent signal.

**Figure 6.**
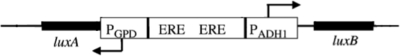
Sanseverino *et al*. placed the alcohol dehydrogenase 1 promoter (P_ADH1_) and the glyceraldehyde-3-phosphate dehydrogenase promoter (P_GPD_) controlling *luxA* and *luxB* under the control of estrogen response elements (ERE) to provide conditional expression only in the presence of estrogenic compounds. Adapted and used with permission from [45].

Using the lessons learned from creation of the bioluminescent yeast strain, work was then begun to develop the eukaryotic *lux* system into a functional bioreporter for the detection of estrogenic compounds—a task that was not possible using prokaryotic hosts. Sanseverino *et al*. [45] built upon the *lux* plasmids developed by Gupta, creating a second set that constitutively expressed the *luxC*, *luxD*, *luxE*, and *frp* genes while regulating expression of the remaining *luxA* and *luxB* genes through insertion of human estrogen response elements (Figure 6). Upon exposure to estrogenic compounds, yeast expressing these regulated *lux* genes would produce a bioluminescent signal in as quickly as 1 h. This improved significantly over the colorimetric yeast estrogen screen, which could take as long as five days to produce results under identical conditions. Within two years of this successful demonstration of *lux*-based bioluminescent yeast as estrogen reporters, the same group had expanded the functionality of the assay by incorporating detection of androgenic compounds as well. This was accomplished by replacing the estrogen response elements that controlled *lux* expression with palindromic androgen response elements. However, this simple change allowed for parallel detection of estrogenic and androgenic compounds when combined with the existing *lux*-based estrogen reporter. Just as before, the *lux*-based system was shown to be more effective, faster, and more specific than existing screening methods [32].

### 3.2. lux Expression in Mammalian Cells

Continuing the development of the *lux* system as a functional eukaryotic reporter system, it was further refined and optimized for expression in human cell lines. This process was undertaken because the *lux* system possesses some unique characteristics compared to the alternative fluorescent and bioluminescent reporter systems that are available for use in human cell lines. Because the *lux* system is capable of producing continuous bioluminescence without addition of an exogenous luciferin, it is the only bioluminescent reporter available for human cell imaging that can function independently, and therefore produce real-time data *in vivo* [7,77]. In addition, because it is bioluminescent rather than fluorescent, it is not subject to the same high levels of background produced by some fluorescent reporters such as GFP [78]. These traits make it an attractive option for imaging in the human cellular background.

Despite the potential benefits of human cell line expression, it would still be five years from the first published report of human optimization until full cassette expression was finally reported [6,7]. Despite the success achieved in expressing the full *lux* cassette in *S. cerevisiae*, it was not possible to transfect human cells with the same constructs and produce a bioluminescent signal. In 2005, Patterson *et al*. [6] were the first to demonstrate that efficient expression of the *luxA* and *luxB* genes required optimization of the DNA sequence of each gene to more closely match the human codon preference. This optimization served to increase the efficiency of transcription/translation and increase the probability that the full length of the open reading frame would be recognized and expressed by the human ribosome (Table 2). These results mirrored those demonstrated in the prokaryotic arena by Craney *et al*. [79], who reported that codon modification of the AT-rich *P. luminescens lux* operon allowed for improved expression in GC-rich bacteria such as *Streptomyces coelicolor*. However, unlike the work of Craney *et al*., Patterson had to include additional modifications such as the replacement of the yeast IRES sequence with a mammalian optimized IRES element to provide for improved expression of the downstream *luxB* gene in the mammalian cellular environment. Through incorporation of these modifications, Patterson *et al*. were able to demonstrate constitutive expression of the *lux* luciferase heterodimer, providing the framework for future optimization of the full *lux* cassette and creating a substrate dependent *lux* reporter system that could be used in a manner similar to firefly luciferase, only with addition of the inexpensive aldehyde n-decanal rather than D-luciferin.

**Figure 7.**
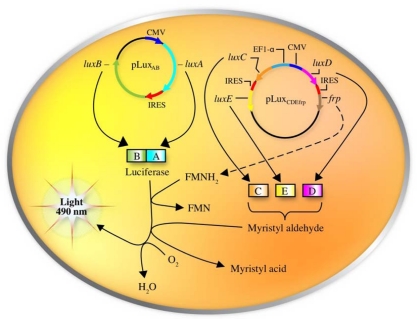
For expression of the full *lux* cassette in a human cell, all of the genes were codon-optimized, separated by IRES elements, and divided across two plasmids that were simultaneously expressed within the host. Adapted and used with permission from [7].

Following the inroads made by Patterson *et al*., it was not long before the full *lux* cassette was optimized for expression in human cell lines. This was accomplished by re-engineering each of the *lux* genes to more closely match the human codon preference, separating them by human optimized IRES elements, and then dividing their expression across three sequences in two separate plasmids. The original construct created by Patterson was retained, but co-expressed with a second plasmid containing the *luxC* and *luxE* genes (separated by an IRES element) under the control of the human elongation factor 1-α promoter and the *luxD* and *frp* genes (also separated by an IRES element) under the control of the cytomegalovirus immediate early promoter (Figure 7).

It is possible that this two plasmid expression system could itself contribute to the production of bioluminescence in human cells since Yagur-Kroll and Belkin [80] have recently reported that splitting the five *lux* genes into two smaller units (*luxAB* and *luxCDE*) resulted in improved bioreporter performance in *E. coli*. This increased bioluminescent production is hypothesized to be due to the associated enhanced transcriptional and/or translational efficiency achieved through the expression of smaller open reading frames, which theoretically could serve the same function in eukaryotic cells as well.

**Figure 8.**
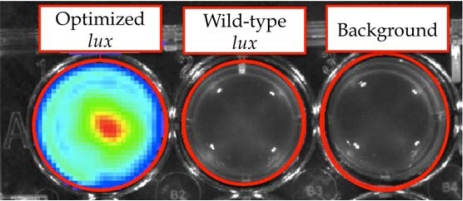
The optimization process employed by Close *et al*. was both necessary and sufficient to induce bioluminescent production from a human cell line upon expression. Adapted and used with permission from [7].

Using this expression strategy, it was demonstrated that these changes were both necessary and sufficient for autonomous production of a bioluminescent signal when expressed in a human kidney cell line (Figure 8) [7]. It should be noted that bioluminescent production from the human-optimized *lux* cassette was demonstrated to be several orders of magnitude lower than that of the more common firefly luciferase reporter and therefore greater numbers of bioluminescent cells were required to produce a significantly detectable signal in both cell culture (15,000 *lux*-expressing cells *vs*. 50 firefly luciferase-expressing cells) and small animal imaging experiments (25,000 *lux*-expressing cells *vs*. 2,500 firefly luciferase cells). However, due to the autonomous nature of the *lux* system, bioluminescent production was maintained over a longer period and produced less variability than did the firefly luciferase system [78].

**Figure 9.**
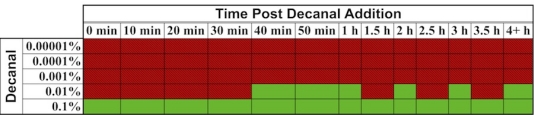
While treatment of constitutively bioluminescent human cells with concentrations of n-decanal at and above 0.001% did not show any significant difference from untreated control cells (red boxes) and all time points surveyed from cells treated with 0.1% n-decanal were significantly diminished in bioluminescent production (green boxes), treatment with 0.01% n-decanal provided the opportunity to view changes in cellular health and metabolism of cells in real time. This type of analysis is made possible because of the unique, autonomous nature of the *lux* reaction. Used with permission from [77].

In one interesting experiment that took advantage of the autonomous nature of *lux* bioluminescence, constitutively bioluminescent human cells expressing the *lux* genes were used to evaluate the cytotoxicity of the aldehyde n-decanal [77]—the same aldehyde that was used by Patterson *et al*. [6] to stimulate bioluminescent production in cell extracts containing optimized *luxA* and *luxB* genes. By monitoring the changes in bioluminescent production following aldehyde treatment, it was possible to evaluate not only which concentrations were cytotoxic to the cells, but also at what time following exposure the effects began to take place, how long the cells were able to continue functioning under a diminished capacity following introduction of the aldehyde, and at what point cells succumbed to treatment and died. It was demonstrated that treatment with 0.00001%, 0.0001%, and 0.001% volumes of aldehyde did not show any changes in bioluminescence [77], despite the fact that this compound has been shown previously to function as a substrate for the *lux* reaction [6]. However, while treatment with a 0.1% volume of aldehyde quickly diminished bioluminescent production, treatment with 0.01% allowed the investigators to visualize a 3.5 h period where the bioluminescent output from cells oscillated between a significant decline and the negative control value, followed by a final, constant departure to significantly reduced bioluminescent production (Figure 9) [77]. The monitoring of these oscillating changes highlights the utility of the autonomous *lux* system, because if individual readings were taken at set time points rather than the near continuous monitoring performed in the investigation, it would have been possible to completely omit this period of activity from the resultant data.

## 4. Future of *lux* Imaging

With the extensive use of *lux* as a bioreporter in bacteria, and the recent proof-in-principal demonstration in eukaryotes, the majority of development that remains for this unique reporter system will most likely revolve around its further optimization and expansion in the eukaryotic cellular background. Close *et al*. [7] have reported that the availability of the FMNH_2_ co-substrate is currently the limiting reagent in the *lux* reaction in human cells, and increases in the efficiency of expressing the Frp protein, which reduces FMN to FMNH_2_, could lead to bioluminescent increases as large as 151-fold. Increases in the availability of the aldehyde co-substrate were also shown to enhance bioluminescent production, however, not to as great an extent and came with possible concerns over increased cytotoxicity. It is also important to note that, unlike alternate reporter systems such as GFP and firefly luciferase, the eukaryotic-optimized *lux* system has only recently become available for use. As the system continues to be used and is exposed to a wider variety of investigators, it can be further optimized and bioluminescent output can be improved. This would enhance the usability of the *lux* system and give it a broader appeal than it has currently.

**Figure 10.**
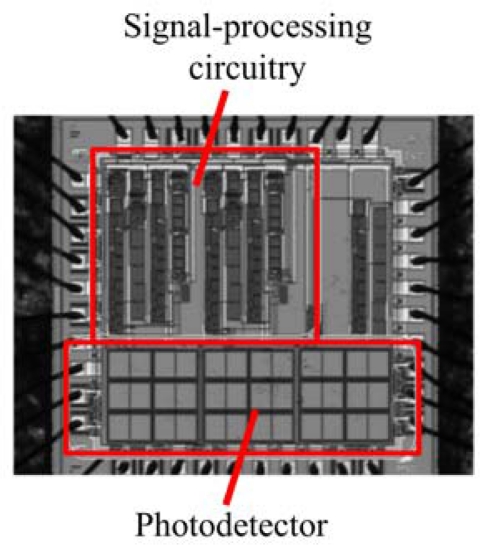
Despite its small size, the BBIC chip contains all of the necessary circuitry for the detection and reporting of bioluminescent cells. By imaging directly on the chip prior to photons passing through host tissue, signal collection will be greatly simplified. Used with permission from [84].

Separate from the biological modifications that could be engineered into the *lux* system, its autonomous nature already presents unique opportunities for merging it directly with existing detection technologies. In several instances, investigators lead by Sayler *et al*. [81–83] have integrated bacteria expressing the full *lux* cassette onto small footprint (∼1 cm^3^), low power (3.3 milliwatt) integrated circuit transducers. These miniaturized devices, called bioluminescent bioreporter integrated circuits (BBICs), can provide for the detection of the bioreporter optical signal, the distinguishing of this signal from noise, the digital processing of the signal, and local communication of the result within a single, self contained package (Figure 10).

When bioluminescent bacterial cells are interfaced to the BBIC, as few as 5,000 can be detected and distinguished from background [81] and it is sensitive enough to differentiate bioluminescent output levels stemming from changes in the concentration of the exposed stimulating analyte [81,82]. These devices could be paired with *lux*-expressing eukaryotic cells and then implanted into small animal models for direct internal imaging of reporter signals without the need to anesthetize or remove the animal from its natural habitat, offering unparalleled opportunities for studying changes in physiology and compound bioavailability under a wide range of conditions. Similarly, these cells could be complexed with microcircuitry capable of initiating hormone or therapeutic compound dosage. In this fashion, the *lux*-expressing cells could be programed to continuously monitor the body for specific target compounds, acting as real-time biosentinels that detect changes in physiology and whose resultant changes in bioluminescent output could trigger the release of counteractive compounds. This would allow the development of fully autonomous, implantable dose/response therapeutic devices.

## 5. Conclusions

While the use of bacterial bioluminescence as a reporter system has been employed for quite a long time, it is still a continually developing reporter system. There are many examples from the recent literature that demonstrate new and creative *lux*-based bacterial biosensors that are employed for myriad sensing applications, and there are also recent examples showing how modification of these genes has expanded their usage to new applications that were previously thought impossible. The unique ability of the *lux* system to produce a bioluminescent signal without exogenous substrate input has ensured that it will continue to find use in basic and applied scientific research for years to come. Whether or not it continues to be improved for function in eukaryotic cells may well decide the true extent of *lux* usage in the future, however, for the time being it remains both an interesting and practical example of the benefits available from an optical reporter system.

## Figures and Tables

**Table 1.** A representative listing of *luxCDABE*-based bioreporters.

**Analyte**	**Time for induction**	**Concentration**	**Reference**
2,3 Dichlorophenol	2 h	50 mg/L	[23]
2,4,6 Trichlorophenol	2 h	10 mg/L	[23]
2,4-D	20–60 min	0.44 mg/L	[26,27]
3-Xylene	Hours	3 μM	[28]
4-Chlorobenzoate	1 h	380 μM–6.5 mM	[29]
4-Nitrophenol	2 h	0.25 mg/L	[23]
Alginate production	1 h	50–150 mM NaCl	[30]
Ammonia	30 min	20 μM	[31]
Androgenic chemicals	3–4 h	10^−9^–10^−10^ M	[32]
Antibiotic effectiveness against *Staphylococcus aureus* infections in mice	4 h	100 CFU	[33]
Antimony (antimonate and antimonite)	3–4 h	0.1 mg/L	[34]
Arsenic	3–4 h	80 μg/L As(V); 8 μg/L As(III)	[35]
BTEX (benzene, toluene, ethylbenzene, xylene)	1–4 h	0.03–50 mg/L	[21]
Cadmium	4 h	19 mg/kg	[36]
Chlorodibromomethane	2 h	20 mg/L	[23]
Chloroform	2 h	300 mg/L	[23]
Chromate	1 h	10 μM	[37]
Cobalt	9 μM	2.0 mM	[38]
Copper	1 h	0.05 mg/L	[39]
Dichloromethane	1–2 h	∼0.01 mg/L	[40]
DNA damage (cumene hydroperoxide)	50 min	6.25 mg/mL	[41]
DNA damage (mitomycin)	1 h	0.032 μg/mL	[42]
	Not specified	0.31 μg/mL	[43]
DNA damage and other cell stressors/activators	A library of *luxCDABE*-based fusions with 689 *E. coli* gene promoters		[44]
Estrogenic chemicals	1 h	10^−11^ M	[45]
Gamma-irradiation	1.5 h	1.5–200 Gy	[46]
Heat shock	20 min	Various, depending on chemical inducer used	[47,48]
Heavy metals	A multi-bioreporter panel for detecting and identifying multiple heavy metal contaminants in a single sample		[49]
Hemolysin production	Not specified	5 mM cAMP	[50]
Hydrogen peroxide	20 min	0.1 mg/L	[51]
*in vivo* monitoring of *Salmonella typhimurium* infections in living mice	4 h	100 CFU	[52]
Iron	Hours	10 nM–1 μM	[53]
Isopropyl benzene	1–4 h	1–100 μM	[54]
Lead	1 h	0.33 mg/L	[39,55]
Mercury	2 h	0.5 ng/L	[56]
*N*-acyl homoserine lactones (3-oxo-C6-HSL)	200 min	3 nM	[57]
Naphthalene	8–24 min	12–120 μM	[58]
Nickel	4–6 h	0.1 μM	[38,59]
Nitrate	∼1 h	1 mg/L	[60]
Organic peroxides	20 min	Not specified	[51]
Oxidative stress	Not specified	0.015 ppm (paraquat)	[61]
PCBs	1–3 h	0.8 μM	[62]
p-chlorobenzoic acid	40 min	0.06 g/L	[29]
p-cymene	<30 min	60 ppb	[63]
Pentachlorophenol	2 h	0.008 mg/L	[23]
Phenol	2 h	16 mg/L	[23]
Salicylate	15 min	36 μM	[58]
Shiga toxin expression in *E. coli*	Gene expression profiling in enterohemorrhagic *E. coli*		[64]
Tetracycline	40 min	5 ng/mL	[65]
Toxicity monitoring	Use of multi-bioreporter arrays to survey and identify multiple chemicals within single samples		[66,67]
Trichloroethylene	1–1.5 h	5–80 μM	[68]
Trinitrotoluene	Not specified	Not specified	[69]
Ultrasound	1 h	500 W/cm^2^	[70]
Ultraviolet light (bacterial)	1 h	2.5–20 J/m^2^	[71]
Ultraviolet light (yeast)	1 h	7 mJ/cm^2^	[72]
Zinc	4 h	0.5–4 μM	[73]

**Table 2.** Transcription and translation prediction scores from the GENSCAN algorithm (http://genes.mit.edu) for expression of wild type (wt) and optimized (op) *luxA* and *luxB* genes in a human host. Score interpretation: 0–50 weak, 50–100 moderate, >100 strong. Used with permission from [6].

**Gene**	**Type**	**Begin**	**End**	**Length**	***I*^a^**	***T*^b^**	**CodRg ^c^**	***P*^d^**	**Translated ^e^**
*luxA* (wt)	1	61	1,083	1,023	45	42	791	0.7	67.01
*luxA* (op)	1	1	1,083	1,083	66	42	1,910	0.88	181.78
*luxB* (wt)	1	1	984	984	51	38	585	0.97	46.37
*luxB* (op)	1	1	984	984	66	41	1,952	0.99	185.60

a Initiaion signal;

b Termination;

c Coding region score;

d Probability of an exon;

e Exon score.

## References

[1] Close D.M., Ripp S., Sayler G.S. (2009). Reporter proteins in whole-cell optical bioreporter detection systems, biosensor integrations, and biosensing applications. Sensors.

[2] McElroy W., Hastings J., Sonnenfeld V., Coulombre J. (1953). The requirement of riboflavin phosphate for bacterial luminescence. Science.

[3] Strehler B., Harvey E., Chang J., Cormier M. (1954). The luminescent oxidation of reduced riboflavin or reduced riboflavin phosphate in the bacterial luciferin-luciferase reaction. Proc. Natl. Acad. Sci. USA.

[4] Belas R., Mileham A., Cohn D., Hilmen M., Simon M., Silverman M. (1982). Bacterial bioluminescence: Isolation and expression of the luciferase genes from *Vibrio harveyi*. Science.

[5] Gupta R.K., Patterson S.S., Ripp S., Sayler G.S. (2003). Expression of the *Photorhabdus luminescens lux* genes (*luxA*, *B*, *C*, *D*, and *E*) in *Saccharomyces cerevisiae*. FEMS Yeast Res.

[6] Patterson S.S., Dionisi H.M., Gupta R.K., Sayler G.S. (2005). Codon optimization of bacterial luciferase (*lux*) for expression in mammalian cells. J. Ind. Microbiol. Biotechnol.

[7] Close D.M., Patterson S.S., Ripp S., Baek S.J., Sanseverino J., Sayler G.S. (2010). Autonomous bioluminescent expression of the bacterial luciferase gene cassette (*lux*) in a mammalian cell line. PLoS One.

[8] Nealson K.H., Hastings J. (1979). Bacterial bioluminescence: Its control and ecological significance. Microbiol. Mol. Biol. Rev.

[9] Meighen E.A. (1991). Molecular biology of bacterial bioluminescence. Microbiol. Rev.

[10] Swartzman E., Silverman M., Meighen E. (1992). The *luxR* gene product of *Vibrio harveyi* is a transcriptional activator of the *lux* promoter. J. Bacteriol.

[11] Engebrecht J., Nealson K., Silverman M. (1983). Bacterial bioluminescence: Isolation and genetic analysis of functions from *Vibrio fischeri*. Cell.

[12] Fernandezpinas F., Wolk C. (1994). Expression of *luxCD-E* in *Anabeaena sp*. can replace the use of exogenous aldehyde for *in vivo* localization of transcription by *luxAB*. Gene.

[13] Frackman S., Anhalt M., Nealson K. (1990). Cloning, organization, and expression of the bioluminescence genes of *Xenorhabdus luminescens*. J. Bacteriol.

[14] Engebrecht J., Simon M., Silverman M. (1985). Measuring gene expression with light. Science.

[15] Belas R., Simon M., Silverman M. (1986). Regulation of lateral flagella gene transcription in *Vibrio parahaemolyticus*. J. Bacteriol.

[16] Shaw J.J., Kado C.I. (1986). Development of a *Vibrio* bioluminescence gene set to monitor phytopathogenic bacteria during the ongoing disease process in a nondisruptive manner. Nat. Biotechnol.

[17] de Weger L., Dunbar P., Mahafee F., Lugtenberg B., Sayler G. (1991). Use of bioluminescence markers to detect *Pseudomonas* spp. in the rhizosphere. Appl. Environ. Microbiol.

[18] Ripp S., Nivens D.E., Ahn Y., Werner C., Jarrell J., Easter J.P., Cox C.D., Burlage R.S., Sayler G.S. (2000). Controlled field release of a bioluminescent genetically engineered microorganism for bioremediation process monitoring and control. Environ. Sci. Technol.

[19] King J.M.H., Digrazia P.M., Applegate B., Burlage R., Sanseverino J., Dunbar P., Larimer F., Sayler G.S. (1990). Rapid, sensitive bioluminescent reporter technology for naphthalene exposure and biodegradation. Science.

[20] Burlage R.S., Sayler G.S., Larimer F. (1990). Monitoring of naphthalene catabolism by bioluminescence with *nah*-*lux* transcriptional fusions. J. Bacteriol.

[21] Applegate B.M., Kehrmeyer S.R., Sayler G.S. (1998). A chromosomally based *tod*-*luxCDABE* whole-cell reporter for benzene, toluene, ethybenzene, and xylene (BTEX) sensing. Appl. Environ. Microbiol.

[22] Abd-El-Haleem D., Ripp S., Scott C., Sayler G.S. (2002). A *luxCDABE*-based bioluminescent bioreporter for the detection of phenol. J. Ind. Microbiol. Biotechnol.

[23] Davidov Y., Rozen R., Smulski D.R., Van Dyk T.K., Vollmer A.C., Elsemore D.A., LaRossa R.A., Belkin S. (2000). Improved bacterial SOS promoter::*lux* fusions for genotoxicity detection. Mutat. Res.

[24] Belkin S., Gu M. (2010). Whole Cell Sensing Systems II: Applications.

[25] Ripp S., Layton A.C., Sayler G.S., Sen K., Ashbolt N.J. (2011). The microbe as a reporter: Microbial bioreporter sensing technologies for chemical and biological detection. Environmental Microbiology: Current Technology and Water Applications.

[26] Hay A.G., Rice J.F., Applegate B.M., Bright N.G., Sayler G.S. (2000). A bioluminescent whole-cell reporter for detection of 2,4-dichlorophenoxyacetic acid and 2,4-dichlorophenol in soil. Appl. Environ. Microbiol.

[27] Toba F.A., Hay A.G. (2005). A simple solid phase assay for the detection of 2,4-D in soil. J. Microbiol. Methods.

[28] Burlage R.S., LaRossa R. (1998). Organic contaminant detection and biodegradation characteristics. Methods in Molecular Biology/Bioluminescence.

[29] Rozen Y., Nejidat A., Gartemann K.H., Belkin S. (1999). Specific detection of *p*-chlorobenzoic acid by *Escherichia coli* bearing a plasmid-borne *fcbA'::lux* fusion. Chemosphere.

[30] Wallace W.H., Fleming J.T., White D.C., Sayler G.S. (1994). An *algD-lux* bioluminescent reporter plasmid to monitor alginate production in biofilms. Microb. Ecol.

[31] Simpson M.L., Paulus M.J., Jellison G.E., Sayler G.S., Nivens D.E., Dionisi H.M., Ripp S., Applegate B., Patterson G., Bolton E. Bioluminescent bioreporter integrated circuits (BBICs): Whole-cell environmental monitoring devices.

[32] Eldridge M.L., Sanseverino J., Layton A.C., Easter J.P., Schultz T.W., Sayler G.S. (2007). *Saccharomyces cerevisiae* BLYAS, a new bioluminescent bioreporter for detection of androgenic compounds. Appl. Environ. Microbiol.

[33] Francis K.P., Joh D., Bellinger-Kawahara C., Hawkinson M.J., Purchio T.F., Contag P.R. (2000). Monitoring bioluminescent *Staphylococcus aureus* infections in mice using a novel *luxABCDE* construct. Infect. Immun.

[34] Flynn H.C., Meharg A.A., Bowyer P.K., Paton G.I. (2003). Antimony bioavailability in mine soils. Environ. Pollut.

[35] Ivask A., Green T., Polyak B., Mor A., Kahru A., Virta M., Marks R. (2007). Fibre-optic bacterial biosensors and their application for the analysis of bioavailable Hg and As in soils and sediments from Aznalcollar mining area in Spain. Biosens. Bioelectron.

[36] Corbisier P., Thiry E., Diels L. (1996). Bacterial biosensors for the toxicity assessment of solid waste. Environ. Toxicol. Water Qual.

[37] Peitzsch N., Eberz G., Nies D.H. (1998). *Alcaligenes eutrophus* as a bacterial chromate sensor. Appl. Environ. Microbiol.

[38] Tibazarwa C., Wuertz S., Mergeay M., Wyns L., van der Lelie D. (2000). Regulation of the *cnr* cobalt and nickel resistance determinant of *Ralstonia eutropha* (*Alcaligenes eutrophus*) CH34. J. Bacteriol.

[39] Riether K.B., Dollard M.A., Billard P. (2001). Assessment of heavy metal bioavailability using *Escherichia coli zntAp::lux* and *copAp::lux* based biosensors. Appl. Microbiol. Biotechnol.

[40] Lopes N., Hawkins S., Jegier P., Menn F.-M., Sayler G., Ripp S. (2012). Detection of dichloromethane with a bioluminescent (*lux*) bacterial bioreporter. J. Ind. Microbiol. Biotechnol.

[41] Belkin S., Smulski D.R., Dadon S., Vollmer A.C., Van Dyk T.K., LaRossa R.A. (1997). A panel of stress-responsive luminous bacteria for the detection of selected classes of toxicants. Wat. Res.

[42] Vollmer A.C., Belkin S., Smulski D.R., Van Dyk T.K., LaRossa R.A. (1997). Detection of DNA damage by use of *Escherichia coli* carrying *recA*'-*lux*, *uvrA*'-*lux*, or *alkA*'-*lux* reporter plasmids. Appl. Environ. Microbiol.

[43] Hwang E.T., Ahn J.M., Kim B.C., Gu M.B. (2008). Construction of a *nrdA::luxCDABE* fusion and its use in *Escherichia coli* as a DNA damage biosensor. Sensors.

[44] van Dyk T.K., DeRose E.J., Gonye G.E. (2001). LuxArray, a high-density, genomewide transcription analysis of *Escherichia coli* using bioluminescent reporter strains. J. Bacteriol.

[45] Sanseverino J., Gupta R.K., Layton A.C., Patterson S.S., Ripp S.A., Saidak L., Simpson M.L., Schultz T.W., Sayler G.S. (2005). Use of *Saccharomyces cerevisiae* BLYES expressing bacterial bioluminescence for rapid, sensitive detection of estrogenic compounds. Appl. Environ. Microbiol.

[46] Min J., Lee C.W., Moon S.H., LaRossa R.A., Gu M.B. (2000). Detection of radiation effects using recombinant bioluminescent *Escherichia coli* strains. Radiat. Environ. Biophys.

[47] Rupani S.P., Gu M.B., Konstantinov K.B., Dhurjati P.S., Van Dyk T.K., Larossa R.A. (1996). Characterization of the stress response of a bioluminescent biological sensor in batch and continuous cultures. Biotechnol. Prog.

[48] van Dyk T.K., Reed T.R., Vollmer A.C., LaRossa R.A. (1995). Synergistic induction of the heat shock response in *Escherichia coli* by simultaneous treatment with chemical inducers. J. Bacteriol.

[49] Jouanneau S., Durand M.J., Courcoux P., Blusseau T., Thouand G. (2011). Improvement of the identification of four heavy metals in environmental samples by using predictive decision tree models coupled with a set of five bioluminescent bacteria. Environ. Sci. Technol.

[50] Bang Y.B., Lee S.E., Rhee J.H., Choi S.H. (1999). Evidence that expression of the *Vibrio vulnificus* hemolysin gene is dependent on cyclic AMP and cyclic AMP receptor protein. J. Bacteriol.

[51] Belkin S., Smulski D.R., Vollmer A.C., Van Dyk T.K., LaRossa R.A. (1996). Oxidative stress detection with *Escherichia coli* harboring a *katG*'::*lux* fusion. Appl. Environ. Microbiol.

[52] Contag C.H., Contag P.R., Mullins J.I., Spilman S.D., Stevenson D.K., Benaron D.A. (1995). Photonic detection of bacterial pathogens in living hosts. Mol. Microbiol.

[53] Khang Y.H., Yang Z.K., Burlage R.S. (1997). Measurement of iron-dependence of *pupA* promoter activity by a *pup*-*lux* bioreporter. J. Microbiol. Biotechnol.

[54] Selifonova O.V., Eaton R.W. (1996). Use of an *ipb*-*lux* fusion to study regulation of the isopropylbenzene catabolism operon of *Pseudomonas putida* RE204 and to detect hydrophobic pollutants in the environment. Appl. Environ. Microbiol.

[55] Magrisso S., Belkin S., Erel Y. (2009). Lead bioavailability in soil and soil components. Water Air Soil Pollut.

[56] Larose C., Dommergue A., Marusczak N., Coves J., Ferrari C.P., Schneider D. (2011). Bioavailable mercury cycling in polar snowpacks. Environ. Sci. Technol.

[57] Yan L., Allen M.S., Simpson M.L., Sayler G.S., Cox C.D. (2007). Direct quantification of N-(3-oxo-hexanoyl)-L-homoserine lactone in culture supernatant using a whole-cell bioreporter. J. Microbiol. Methods.

[58] Heitzer A., Webb O.F., Thonnard J.E., Sayler G.S. (1992). Specific and quantitative assessment of naphthalene and salicylate bioavailability by using a bioluminescent catabolic reporter bacterium. Appl. Environ. Microbiol.

[59] Everhart J.L., McNear D., Peltier E., van der Lelie D., Chaney R.L., Sparks D.L. (2006). Assessing nickel bioavailability in smelter-contaminated soils. Sci. Total Environ.

[60] Abd-El-Haleem D., Ripp S., Zaki S., Sayler G.S. (2007). Detection of nitrate/nitrite bioavailability in wastewater using a *luxCDABE*-based *Klebsiella oxytoca* bioluminescent bioreporter. J. Microbiol. Biotechnol.

[61] Lee H.J., Gu M.B. (2003). Construction of a *sodA::luxCDABE* fusion *Escherichia coli*: Comparison with a *katG* fusion strain through their responses to oxidative stresses. Appl. Microbiol. Biotechnol.

[62] Layton A.C., Muccini M., Ghosh M.M., Sayler G.S. (1998). Construction of a bioluminescent reporter strain to detect polychlorinated biphenyls. Appl. Environ. Microbiol.

[63] Ripp S., Applegate B., Nivens D.E., Simpson M.L., Sayler G.S. Advances in whole-cell bioluminescent bioreporters for environmental monitoring and chemical sensing.

[64] Shimizu T., Ohta Y., Tsutsuki H., Noda M. (2011). Construction of a novel bioluminescent reporter system for investigating Shiga toxin expression of enterohemorrhagic *Escherichia coli*. Gene.

[65] Hansen L.H., Sorensen S.J. (2000). Detection and quantification of tetracyclines by whole cell biosensors. FEMS Microbiol. Lett.

[66] Elad T., Benovich E., Magrisso S., Belkin S. (2008). Toxicant identification by a luminescent bacterial bioreporter panel: Application of pattern classification algorithms. Environ. Sci. Technol.

[67] Elad T., Lee J.H., Gu M.B., Belkin S., Belkin S., Gu M.B. (2010). Microbial cell arrays. Whole Cell Sensing Systems I: Reporter Cells and Devices.

[68] Shingleton J.T., Applegate B.M., Nagel A.C., Bienkowski P.R., Sayler G.S. (1998). Induction of the *tod* operon by trichloroethylene in *Pseudomonas putida* TVA8. Appl. Environ. Microbiol.

[69] Burlage R.S., Patek D.R., Everman K.R. Method for Detection of Buried Explosives Using a Biosensor.

[70] Vollmer A.C., Kwakye S., Halpern M., Everbach E.C. (1998). Bacterial stress response to 1-megahertz pulsed ultrasound in the presence of microbubbles. Appl. Environ. Microbiol.

[71] Elasri M.O., Miller R.V. (1998). A *Pseudomonas aeruginosa* biosensor responds to exposure to ultraviolet radiation. Appl. Microbiol. Biotechnol.

[72] Bakhrat A., Eltzov E., Finkelstein Y., Marks R.S., Raveh D. (2011). UV and arsenate toxicity: A specific and sensitive yeast bioluminescence assay. Cell Biol. Toxicol.

[73] Erbe J.L., Adams A.C., Taylor K.B., Hall L.M. (1996). Cyanobacteria carrying an *smt-lux* transcriptional fusion as biosensors for the detection of heavy metal cations. J. Ind. Microbiol.

[74] Almashanu S., Musafia B., Hadar R., Suissa M., Kuhn J. (1990). Fusion of *luxA* and *luxB* and its expression in *Escherichia coli*, *Saccharomyces cerevisiae* and *Drosophila melanogaster*. J. Biolumin. Chemilumin.

[75] Kirchner G., Roberts J.L., Gustafson G.D., Ingolia T.D. (1989). Active bacterial luciferase from a fused gene: Expression of a *Vibrio harveyi luxAB* translational fusion in bacteria, yeast and plant cells. Gene.

[76] Olsson O., Koncz C., Szalay A.A. (1988). The use of the *luxA* gene of the bacterial luciferase operon as a reporter gene. Mol. Gen. Genet.

[77] Close D., Hahn R., Ripp S., Sayler G.S. Determining toxicant bioavailability using a constitutively bioluminescent human cell line.

[78] Close D.M., Hahn R., Patterson S.S., Ripp S., Sayler G.S. (2011). Comparison of human optimized bacterial luciferase, firefly luciferase, and green fluorescent protein for continuous imaging of cell culture and animal models. J. Biomed. Opt.

[79] Craney A., Hohenauer T., Xu Y., Navani N.K., Li Y.F., Nodwell J. (2007). A synthetic *luxCDABE* gene cluster optimized for expression in high-GC bacteria. Nucleic Acids Res.

[80] Yagur-Kroll S., Belkin S. (2011). Upgrading bioluminescent bacterial bioreporter performance by splitting the *lux* operon. Anal. Bioanal. Chem.

[81] Nivens D.E., McKnight T.E., Moser S.A., Osbourn S.J., Simpson M.L., Sayler G.S. (2004). Bioluminescent bioreporter integrated circuits: Potentially small, rugged and inexpensive whole-cell biosensors for remote environmental monitoring. J. Appl. Microbiol.

[82] Ripp S., DiClaudio M., Sayler G., Mitchell R., Gu J. (2010). Biosensors as environmental monitors. Environmental Microbiology.

[83] Vijayaraghavan R., Islam S., Zhang M., Ripp S., Caylor S., Bull N.D., Moser S., Terry S., Blalock B., Sayler G. (2007). A bioreporter bioluminescent integrated circuit for very low-level chemical sensing in both gas and liquid environments. Sens. Actuat. B-Chem.

[84] Simpson M.L., Sayler G.S., Applegate B.M., Ripp S., Nivens D.E., Paulus M.J., Jellison G.E. (1998). Bioluminescent-bioreporter integrated circuits form novel whole-cell biosensors. Trends Biotechnol.

